# Halvade-RNA: Parallel variant calling from transcriptomic data using MapReduce

**DOI:** 10.1371/journal.pone.0174575

**Published:** 2017-03-30

**Authors:** Dries Decap, Joke Reumers, Charlotte Herzeel, Pascal Costanza, Jan Fostier

**Affiliations:** 1 Department of Information Technology, IDLab, Ghent University - imec, Ghent, Belgium; 2 Janssen Research & Development, a division of Janssen Pharmaceutica N.V., Beerse, Belgium; 3 Imec, Leuven, Belgium; 4 Intel Corporation Belgium, Leuven, Belgium; 5 ExaScience Life Lab, Leuven, Belgium; Tianjin University, CHINA

## Abstract

Given the current cost-effectiveness of next-generation sequencing, the amount of DNA-seq and RNA-seq data generated is ever increasing. One of the primary objectives of NGS experiments is calling genetic variants. While highly accurate, most variant calling pipelines are not optimized to run efficiently on large data sets. However, as variant calling in genomic data has become common practice, several methods have been proposed to reduce runtime for DNA-seq analysis through the use of parallel computing. Determining the effectively expressed variants from transcriptomics (RNA-seq) data has only recently become possible, and as such does not yet benefit from efficiently parallelized workflows. We introduce Halvade-RNA, a parallel, multi-node RNA-seq variant calling pipeline based on the GATK Best Practices recommendations. Halvade-RNA makes use of the MapReduce programming model to create and manage parallel data streams on which multiple instances of existing tools such as STAR and GATK operate concurrently. Whereas the single-threaded processing of a typical RNA-seq sample requires ∼28h, Halvade-RNA reduces this runtime to ∼2h using a small cluster with two 20-core machines. Even on a single, multi-core workstation, Halvade-RNA can significantly reduce runtime compared to using multi-threading, thus providing for a more cost-effective processing of RNA-seq data. Halvade-RNA is written in Java and uses the Hadoop MapReduce 2.0 API. It supports a wide range of distributions of Hadoop, including Cloudera and Amazon EMR.

## Introduction

Recently, a number of methods have been introduced to accelerate read mapping and variant calling through the use of parallel and distributed computing techniques: HugeSeq [1], MegaSeq [2], Churchill [3] and Halvade [4] implement a DNA-seq variant calling pipeline according to the Best Practices recommendations [5] for use with the GATK [6, 7] variant caller. These tools exploit the fact that read mapping is parallel by read, i.e., aligning one read is independent of the alignment of other reads, while variant calling is parallel by genomic region, i.e., variant calling in a certain genomic region is independent of variant calling in other regions. As such, the runtime to process whole genome or whole exome sequencing data sets is strongly reduced. Other parallel DNA-seq variant calling pipelines that do not rely on GATK include SpeedSeq [8] and ADAM [9].

Nowadays, RNA-seq datasets are becoming increasingly available. Even though primarily intended to identify transcripts and quantify expression, RNA-seq data can equally be used to call single nucleotide variants [10]. There are two main conceptual differences with DNA-seq based variant calling. First, the mapping step needs to be modified to avoid false positive variant calling at exon-exon junctions, by using a sample-specific genome index containing junction information. Second, as the assumptions on coverage depth and allelic balance used in DNA-seq based variant calling do not hold in RNA-seq, where coverage depth is dependent on transcript expression, and allele-specific expression can influence allelic balance, the variant caller should be adjusted accordingly. For that purpose, the GATK Best Practices recommendations have been adapted to involve two passes of STAR [11] for spliced read alignment, Picard (http://picard.sourceforge.net/) for data preprocessing and GATK for variant calling. Processing a typical RNA-seq sample using this pipeline on a single CPU core takes ∼28 hours. Enabling multi-threading on a 20-core machine reduces runtime only by a factor of two, which indicates considerable loss of performance during execution.

Here we present Halvade-RNA, a parallel framework for variant calling from RNA-seq data that relies on the MapReduce programming model [12]. MapReduce has previously been used in bioinformatics for different applications [13–15]. Rather than relying on multi-threading, Halvade-RNA runs several instances of the different tools involved (STAR, Picard, GATK) in parallel on subsets of the data, resulting in more efficient use of resources and thus lower cost of computing. In addition to reducing the runtime of a single RNA-seq sample, Halvade-RNA accelerates batch processing of multiple RNA-seq samples on any compute infrastructure on which Hadoop/MapReduce is installed, including public cloud platforms such as Amazon Web Services. To the best of our knowledge, this is the first framework to accelerate variant calling pipelines for RNA-seq data. The source is available at http://bioinformatics.intec.ugent.be/halvade under GPL license.

## Materials and methods

### RNA-seq variant calling pipeline

In this work, we adopt the GATK RNA-seq variant calling pipeline as described in https://software.broadinstitute.org/gatk/guide/article?id=3891. Table 1 lists the different steps involved.

**Table 1:** RNA-seq variant calling pipeline used in this work.

Step	Tool	Input	Output
Read mapping (1^st^ pass)	STAR	FASTQ + index	SAM + splice junctions
Rebuild genome index	STAR	ref. genome + splice junctions	new index
Read mapping (2^nd^ pass)	STAR	FASTQ + new index	SAM
Add readgroups and sort	Picard	SAM	BAM
Mark duplicates	Picard	BAM	BAM
Split ‘N’ Trim	GATK	BAM	BAM
Indel realignment	GATK	BAM	BAM
Base quality score recalibration	GATK	BAM	BAM
Variant calling	GATK	BAM	VCF

In order to obtain accurate spliced read alignment, a two-step approach using the STAR aligner is used as first described in [16]. During the first pass, spliced alignment is performed without prior knowledge of splice sites. Identified splice junctions are then incorporated in a new genome index file which is subsequently used to guide the final alignments during the second pass. Next, Picard is used to add readgroup information, sort the aligned records according to genomic position, mark read duplicates and convert the SAM file to binary BAM format. The GATK Split‘N’Trim module is used to split reads into different exon segments and trim reads that overlap with intronic regions. Reads that contain short insertions or deletions (indels) are realigned to avoid false positive variant calls in later steps. Additionally, the per-base quality scores are recalibrated to accommodate certain batch artifacts. Finally, variants are called using the HaplotypeCaller and written to VCF file.

### The RNA pipeline in MapReduce

**Fig 1:**
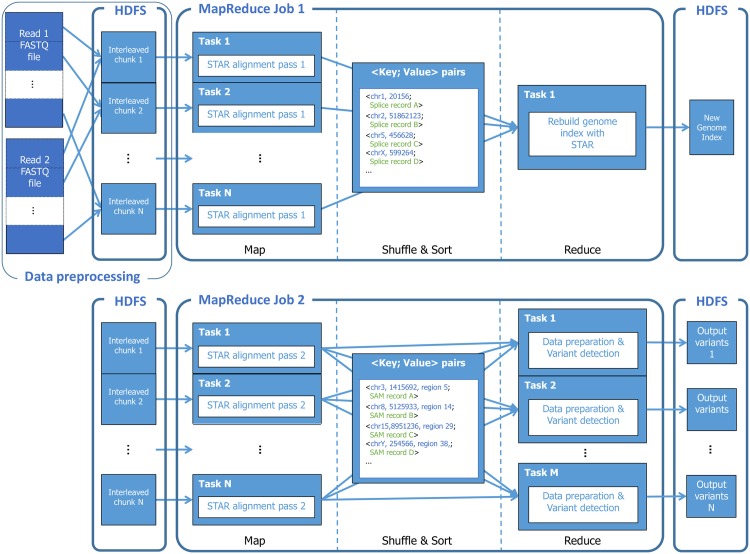
Overview of the RNA-seq pipeline in Halvade-RNA. In the first job, reads are aligned in parallel in order to identify splice junctions and the reference genome index is rebuilt using this information. In the second job, final alignments are produced and after sorting and grouping the aligned reads by genomic region, the different Picard and GATK steps are executed in parallel.

Because the RNA-seq variant calling pipeline involves two passes of the STAR aligner, it cannot be readily expressed in the Halvade MapReduce framework as implemented for DNA-seq variant calling [4]. Whereas the DNA-seq variant calling pipeline could be implemented using a single MapReduce job, two MapReduce jobs are required for Halvade-RNA. Fig 1 provides an overview of the framework.

First, the input FASTQ files are interleaved (such that paired-end reads are adjacent to each other) and split into smaller file chunks. During the map phase of the first MapReduce job, these input chunks are processed in parallel by multiple instances of the STAR aligner. To avoid loading the reference genome index from disk by each STAR instance individually, the genome index is first loaded in shared memory, after which all the STAR instances on this node can access this genome index from RAM. During this first alignment pass, the actual read alignments (i.e., the SAM records) are ignored and only splice junction information is retained. For each read that spans multiple exons, STAR produces a record containing junction information, such as genomic location and strand. The Map tasks emit this information as intermediate <key, value> pairs, with the key holding tuples of integers containing the contig index and the position of the splice junction, and the value containing the STAR-generated string with splice junction information. When all Map tasks are finished, all intermediate <key, value> pairs are sent to a single reducer and written to file. In the reduce task, this file is subsequently used by STAR to build a new genome index that incorporates this slice junction information. This is a purely sequential step, which should be kept as short as possible. We therefore configure STAR to build a sparse index, where only a fraction of the genomic locations are indexed. While using a sparse index results in a slightly higher runtime during the second alignment phase, the reduced runtime during index construction (5 min for a typical RNA-seq sample, as opposed to approximately 30 min for a dense index) ensures the lowest overall runtime.

The second MapReduce job is similar to the DNA-seq variant calling MapReduce implementation and we refer to [4] for more details. RNA-seq reads are again aligned in parallel during the Map phase, using the newly constructed genome index. The Map tasks emit <key, value> pairs where the value represents an actual SAM record and the key a composite structure that contains the genomic location to which the read aligns. In between Map and Reduce phases, the intermediate <key, value> pairs are sorted in parallel according to genomic location by the MapReduce framework in a highly efficient manner. This step replaces the sorting functionality otherwise achieved by Picard. The sorted SAM records are converted to BAM format using Hadoop-BAM [17] and partitioned according to a user-specified number of genomic regions.

During the Reduce phase, remaining data preprocessing and variant calling steps are performed in parallel through the concurrent processing of multiple genomic regions. To achieve this, multiple instances of Picard and GATK are run in parallel, each instance operating on a distinct genomic region. These steps are similar for the DNA-seq variant calling pipeline. The only notable difference is the addition of the Split‘N’Trim module. Called variants are written to VCF files, one VCF file per reduce task. Optionally, these partial VCF files can be merged into a single VCF file in a third, lightweight MapReduce job. It should be noted that Halvade-RNA can also be executed on existing BAM files. In that scenario, the first MapReduce job is skipped and the Map phase of the second job is modified in order to partition the provided BAM file.

As RNA-seq analysis often involves the quantification of gene expression, Halvade-RNA provides the option to count the number of reads per exon. This is done by the FeatureCounts tool [18] and is run per Reduce task, and thus in parallel per genomic region. Again, optionally, the counts per genomic region can be merged into a single file using an additional lightweight MapReduce job.

Finally we remark that the used tools are still often improved and features are added, so it is important to note that Halvade-RNA allows replacing the binaries of the tools with newer versions, assuming that the command line arguments remain the same. Similarly, new tools could easily be added by updating the source code and calling the new tools appropriately.

### Benchmark setup

Halvade-RNA was benchmarked on 9 RNA-seq samples (SNU-1033, SNU-1041, SNU-1214, SNU-213, SNU-216, SNU-308, SNU-489, SNU-601, SNU-668) from the Cancer Cell Line Encyclopedia [19], each sample containing approximately 175 million 101 bp paired-end reads originating from a poly(A) selection experiment. The benchmarks were run using Halvade-RNA version 1.2.0, implemented using STAR version 2.4.0h1, Picard version 1.112, GATK version 3.4 (nightly-2015-05-12-gcdf54f8) and Java version 1.7.0, run on a Cloudera distribution based on Apache Hadoop (CDH) 5.0.0 with Hadoop version 2.3.0. Note that Halvade-RNA has been validated for compatibility with the more recent Hadoop version 2.6.0 (CDH version 5.10.0), Java version 1.8.0 and GATK version 3.7. We used a two-node cluster, each node containing 20 CPU cores (dual-socket Intel Xeon E5-2660 v3 @ 2.60GHz) and 128 GByte of RAM. The nodes were interconnected by an FDR Infiniband network. Halvade-RNA was configured to run 10 parallel instances of STAR per node (2 threads per instance) and 20 parallel instances (single-threaded) of Picard and GATK per node. This way, all available CPU cores are used.

Additionally, Halvade-RNA was compared with GNU parallel [20] for multi-sample processing of all 9 samples. Halvade-RNA was run with identical configuration as described above. GNU parallel was configured to run per node a single instance of STAR using all available CPU cores for multi-threading, as STAR has very good multi-threading capabilities but uses up to 40 GByte of RAM per instance. Because the BAM processing and variant calling steps achieve poor speedups with multi-threading, GNU parallel was configured to run multiple parallel instances of GATK and Picard, each instance processing a different sample and using two threads (only for GATK).

## Results & discussion

### Parallel performance

Four cases were set up to assess the performance of Halvade-RNA: i) the original RNA-seq variant calling pipeline on a single CPU core, ii) the original RNA-seq variant calling pipeline on 20 CPU cores, with multi-threading enabled in both STAR and GATK, iii) the Halvade-RNA pipeline on the same 20-core machine, and iv) the Halvade-RNA pipeline on two 20-core machines (Table 2). For the original pipeline, the obtained speedup using multi-threading is only 2.16 on average (min: 2.03, max: 2.54). In contrast, Halvade-RNA shows a speedup of 9.18 on average (min: 7.65, max: 9.95) on the same node, indicating that Halvade-RNA is on average 4.25 times faster when identical compute resources are used. Halvade-RNA relies primarily on multi-tasking rather than multi-threading, and measuring the average runtimes and speedups per phase of the pipeline (Table 3) shows that especially for the variant calling steps this proves to be more efficient. As such, Halvade-RNA not only reduces analysis time but also substantially reduces the financial cost for computing. Using two nodes, the average parallel speedup increases to 13.72 (min: 9.56, max: 16.26).

**Table 2:** Benchmarks of the RNA-seq variant calling pipeline per sample.

	Runtime for sample (speedup)			
	Classical pipeline		Halvade-RNA pipeline	
Sample	1 node × single core	1 node × 20 cores	1 node × 20 cores	2 nodes × 20 cores
SNU-1033	26h 6min (n/a)	12h 53min (2.03×)	3h 25min (7.65×)	2h 44min (9.56×)
SNU-1041	27h 48min (n/a)	12h 51min (2.16×)	3h 3min (9.09×)	1h 48min (15.46×)
SNU-1214	34h 40min (n/a)	13h 38min (2.54×)	3h 33min (9.77×)	2h 23min (14.56×)
SNU-213	27h 11min (n/a)	12h 59min (2.09×)	2h 50min (9.61×)	1h 44min (15.66×)
SNU-216	27h 21min (n/a)	13h 1min (2.10×)	2h 46min (9.87×)	1h 44min (15.81×)
SNU-308	27h 48min (n/a)	13h 25min (2.07×)	2h 48min (9.95×)	1h 43min (16.26×)
SNU-489	27h 10min (n/a)	12h 30min (2.17×)	3h 8min (8.69×)	2h 16min (11.98×)
SNU-601	26h 48min (n/a)	12h 59min (2.07×)	2h 59min (9.01×)	2h 5min (12.91×)
SNU-668	25h 59min (n/a)	12h 7min (2.14×)	2h 49min (9.24×)	1h 52min (13.97×)
average	27h 52min (n/a)	12h 56min (2.16×)	3h 2min (9.18×)	2h 2min (13.72×)

**Table 3:** Average runtimes per phase of the RNA-seq pipeline.

		Runtime and speedup per phase			
Pipeline	No. of nodes and cores	Pass 1 map	Rebuild genome	Pass 2 map	Variant calling steps
Classical pipeline	1 node × single core	1h 19min (n/a)	4min (n/a)	3h 29min (n/a)	23h 1min (n/a)
	1 node × 20 cores	6min (14.24×)	2min (2.18×)	22min (9.69×)	12h 27min (1.85×)
Halvade-RNA	1 nodes × 20 cores	14min (5.69×)	4min (1.02×)	39min (5.29×)	2h 3min (11.21×)
	2 nodes × 20 cores	8min (9.29×)	4min (1.01×)	22min (9.49×)	1h 26min (16.04×)

Note that similar results were obtained on a public cloud platform (Amazon EMR). Using a single node of the type r3.8xlarge, we obtain an average execution time of 3h 29 min per sample (min: 3h 4 min, max: 4h 28 min). Using two nodes, the average runtime decreases to 2h 32 min per sample (min: 2h 1 min, max: 3h 46 min). The Amazon nodes have only 16 CPU cores and an additional data transfer from central S3 storage to the local worker nodes storage is required which explains the slightly higher runtimes. The average financial cost per sample was 14.15 US dollar when using a single node and 20.48 US dollar when using two nodes (pricing of December 2016).

### Multi-sample throughput

**Fig 2:**
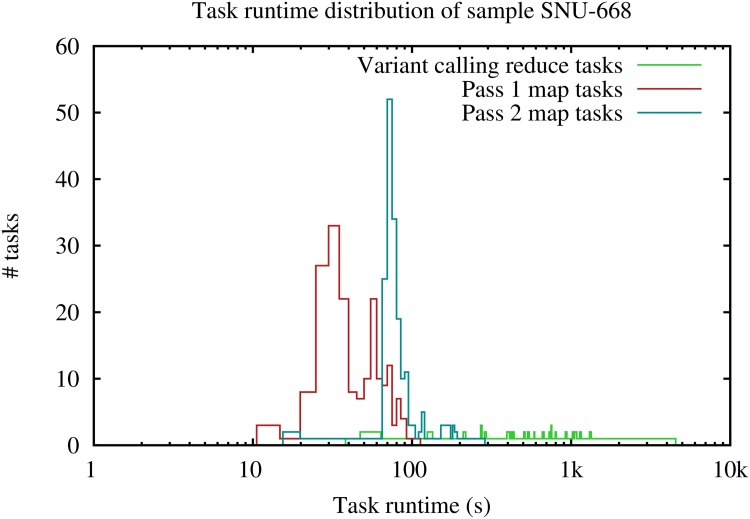
Runtime distribution of Map and Reduce tasks of both jobs. Note that the average runtime of the pass 2 map phase increases slightly due to the sparse index. Also note that the Reduce phase of the first job is not displayed as this comprises only a single job.

Given the large variability in gene expression within one RNA-seq sample, certain genomic chunks can have significantly more aligned reads. For example, in our experiment, coverage depth between genomic chunks could vary as much as thousand fold. As a consequence, there is large variability in execution time during the reduce phases, making it more difficult to balance load than in the Halvade-DNA framework, as coverage depth is more uniform in DNA-seq data. Fig 2 shows the distribution of the runtimes of the MapReduce tasks for sample SNU-668, other samples have similar distributions. The map phases of both MapReduce jobs show a clear peak, with the slight shift for the second map phase resulting from the use of a sparse genome index. The variant calling reduce tasks show a very wide range of runtimes, ranging from about a minute for the fastest task up to one hour for the slowest task. Furthermore, rebuilding the STAR genome (a sequential step) is a second source of losing computing resources. In a realistic scenario, the available compute infrastructure will be used to process multiple samples. In that case, MapReduce jobs can be overlapped by using idle slots to start processing the next sample, even though the current sample is not yet fully completed. This way of working minimizes idle time and thus increases throughput. Table 4 lists the total runtimes for processing all 9 samples. As a reference to calculate the overall speedup, we use the sum of all runtimes on a single-threaded pipeline, which is ∼251 hours. The multi-sample batch processing script (GNU parallel) for the original pipeline processes the 9 samples in 40h 7min with 20 CPU cores on a single node. Using two nodes, we distribute the samples over the two nodes and run the sequential pipeline and the batch script for 4 and 5 samples per node (adjusted to run with 24GB and 4 cores per sample in the GNU parallel steps), resulting in a total runtime of 21h 3min. Using Halvade-RNA in batch mode, a total runtime of 22h 17min is obtained using a single node and a total runtime of 11h 48min on two nodes. This is respectively ∼4h and ∼6.5h faster compared to the sum of the per-sample runtimes. Clearly, the ability to avoid idle time again significantly increases the efficient use of compute resources.

**Table 4:** Runtime for the batch processing of all 9 RNA-seq samples.

Pipeline	No. of nodes and cores	Sum of per-sample runtime (speedup)	Batch processing runtime (speedup)
Classical pipeline	1 node × single core	250h 52min (n/a)	250h 52min (n/a)
	1 node × 20 cores	116h 24min (2.16×)	40h 7min (6.25×)
	2 node × 20 cores	63h 21m (3.96×)	21h 3min (11.92×)
Halvade-RNA	1 nodes × 20 cores	27h 20min (9.18×)	22h 17min (11.26×)
	2 nodes × 20 cores	18h 17min (13.72×)	11h48min(21.26×)

### Quality assessment

**Fig 3:**
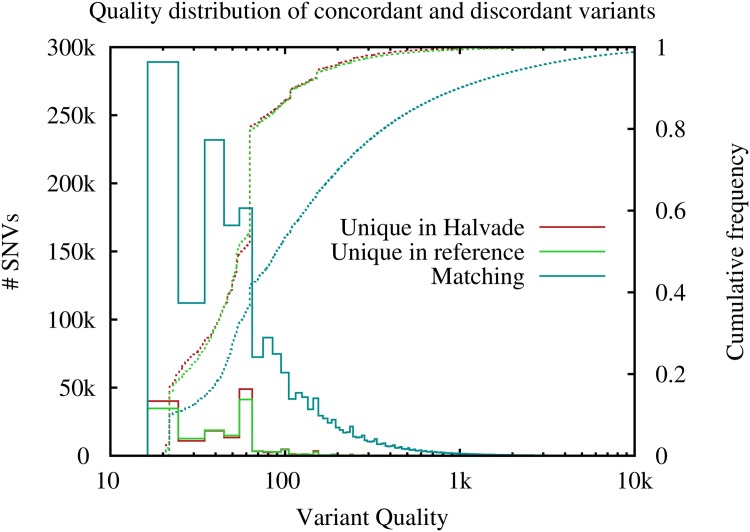
Comparison of variant Quality between Halvade and the reference pipeline. Shows the quality distribution of all variants taken from all 9 samples.

Finally, we show the per-sample concordance in the variants called by the original sequential pipeline and Halvade-RNA (Table 5). On average, 93.8% of the variants found by the sequential pipeline are also called by Halvade-RNA. Variants that are called in either only the sequential pipeline or only the Halvade-RNA pipeline are supported by fewer reads and have a ∼8-fold lower average quality score compared with the overlapping variants, and are thus more likely to be filtered from a high-quality variant list. The normalized distribution of the variant quality for each of the three subsets, matching variants and variants unique to either Halvade or the original pipeline, is shown in Fig 3. The origin of these discordant variants lies in the variability during the read mapping step. Typically these variants are located in regions that are part of repeating patterns, causing the reads to align to multiple locations. Either in the parallelized or in the original sequential pipeline, variants residing in these regions have a high probability of being false positive calls.

**Table 5:** Per sample overlap and average quality score.

Sample	Overlapping variants(%)	Avg. qual score overlapping variants	Avg. qual score Halvade-unique variants	Avg. qual score reference-unique variants
SNU-1033	93.9	651.6	80.3	92.4
SNU-1041	93.4	803.2	85.6	92.7
SNU-1214	93.4	741.3	82.6	92.7
SNU-213	94.3	612.7	74.0	87.2
SNU-216	94.2	660.8	83.7	94.1
SNU-308	93.4	522.7	71.5	71.8
SNU-489	94.4	773.4	81.9	98.3
SNU-601	93.3	742.1	94.4	116.4
SNU-668	93.9	671.0	73.9	88.0

Average per-sample variant quality score (QUAL) for i) variants called by both the single-threaded pipeline and Halvade-RNA on 2 nodes × 20 cores, ii) variants called only by Halvade-RNA (‘Halvade-unique’), iii) variants called only by the single-threaded pipeline (‘reference-unique’).

## Conclusion

We have implemented a parallelized variant calling pipeline for RNA-seq data using a MapReduce approach, and compared the efficiency and accuracy of the pipeline to the original sequential implementation. Running the original pipeline using a single core requires on average 27.9 hours per sample. When enabling multi-threading on 20 CPU cores in STAR and GATK, the average runtime per sample decreases to 12.9 hours, largely due to the poor scaling behavior of GATK. In contrast, on the same node, when using Halvade-RNA configured to run 10 parallel instances of STAR (2 threads per instance) and 20 parallel instances (single threaded) of Picard and GATK, average runtime decreases to ∼3 hours, corresponding to a parallel speedup of 9.18 over sequential execution of the pipeline. Clearly, the use of Halvade on a single node strongly reduces average runtime and results in a more cost-effective use of the compute resources. On two nodes, the average runtime further decreases to ∼2 hours.

Obtaining a good load balance across parallel tasks is challenging for RNA-seq data, given the large variability in gene expression –and thus coverage depth–across genomic regions. To minimize idle time introduced by slower jobs, Halvade-RNA can be operated in batch mode. In that case, idle slots can be used to start processing the next sample even though the current sample is not yet fully completed. Processing all of the 9 samples in batch mode on the two-node cluster yields a total runtime of 11.8 hours. In comparison, running the pipeline in batch mode gives a runtime of 21h on a two-node cluster. Even though batch mode does not decrease the per-sample processing time below 2 hours, it considerably increases the overall throughput through a more efficient use of compute infrastructure.

On average, variants identified by Halvade-RNA and the sequential pipeline have a 93.8% overlap. The variability is almost exclusively caused by variability during read mapping. Variants called by either only Halvade-RNA or the sequential pipeline have a much lower number of supporting reads and hence correspond to low-confidence variants.
